# Population Genetic Differentiation and Taxonomy of Three Closely Related Species of *Saxifraga* (Saxifragaceae) from Southern Tibet and the Hengduan Mountains

**DOI:** 10.3389/fpls.2017.01325

**Published:** 2017-07-28

**Authors:** Qing-Bo Gao, Yan Li, Zhuo-Ma Gengji, Richard J. Gornall, Jiu-Li Wang, Hai-Rui Liu, Liu-Kun Jia, Shi-Long Chen

**Affiliations:** ^1^Key Laboratory of Adaptation and Evolution of Plateau Biota, Northwest Institute of Plateau Biology, Chinese Academy of Sciences Xining, China; ^2^Key Laboratory of Crop Molecular Breeding of Qinghai Province, Northwest Institute of Plateau Biology, Chinese Academy of Sciences Xining, China; ^3^University of Chinese Academy of Sciences Beijing, China; ^4^Department of Genetics, University of Leicester Leicester, United Kingdom

**Keywords:** *Saxifraga*, the Hengduan Mountains, allopatric speciation, vicariance, taxonomic suggestion

## Abstract

The effects of rapid, recent uplift of the Hengduan Mountains on evolution and diversification of young floristic lineages still remain unclear. Here, we investigate diversification of three closely related *Saxifraga* species with a distribution restricted to the Hengduan Mountains (HM) and southern Tibet, and comment on their taxonomy based on molecular evidence. Three chloroplast DNA fragments (*rbcL, trnL-F, trnS-G*) and the nuclear ribosomal DNA internal transcribed spacer (ITS) were employed to study genetic structure across 104 individuals from 12 populations of *Saxifraga umbellulata, S. pasumensis*, and *S. banmaensis*. Chloroplast DNA (cpDNA) phylogenies revealed two well supported clades, corresponding to *S. umbellulata* and *S. pasumensis* plus *S. banmaensis*. Topology of the ITS phylogeny was largely congruent with that generated from cpDNA haplotypes, but with minor conflicts which might be caused by incomplete lineage sorting. Analyses of molecular variance of both cpDNA and ITS datasets revealed that most variation was held between *S. pasumensis* s.l. (with *S. banmaensis*) and *S. umbellulata* (92.31% for cpDNA; 69.78% for ITS), suggesting a high degree of genetic divergence between them. Molecular clock analysis based on ITS dataset suggested that the divergence between *S. pasumensis* s.l. and *S. umbellulata* can be dated to 8.50 Ma, probably a result of vicariant allopatric diversification associated with the uplift events of the HM. Vicariance associated with HM uplifts may also have been responsible for infraspecific differentiation in *S. pasumensis*. In contrast, infraspecific differentiation in *S. umbellulata* was most likely triggered by Quaternary glaciations. The much lower levels of gene diversity within populations of *S. pasumensis* compared with *S. umbellulata* could have resulted from both range contractions and human collection on account of its putative medicinal properties. Combining evidence from morphology, geographical distributions and molecular phylogenetic data, we recommend that *S. banmaensis* should be treated as a synonym of *S. pasumensis* which in turn, and based on the same sources of evidence, should be treated as a separate species rather than as a variety of *S. umbellulata*.

## Introduction

High levels of biodiversity are commonly associated with mountains as reported for the Caucasus, the Andes, the Himalayas and the Hengduan Mountains (Myers et al., 2000; Hoorn et al., 2013; Boufford, 2014; Hughes, 2016). Origin of mountainous biodiversity is complex and could include immigration of preadapted lineages (e.g., Johansson et al., 2007; Price et al., 2014; Hughes and Atchison, 2015), *in situ* diversification (Hughes and Eastwood, 2006; Pennington et al., 2010; Lagomarsino et al., 2016), persistence of ancestral lineages (lower extinction rate; Lancaster and Kay, 2013), or a combination of them (e.g., Ebersbach et al., 2017a). Compared to other mountains such as the Andes, evolution and diversification of organisms in the Qinghai-Tibetan Plateau (QTP) and its surrounding mountains remains poorly understood (Favre et al., 2015), mainly because of a lack of a consensus scenario on the geological history of this region. Contrasting opinions on the timing and processes of QTP uplift, such as recent abrupt uplift (e.g., Li et al., 1995; Shi et al., 1998; An et al., 2001) vs. ancient progressive uplift (e.g., Wang et al., 2008), have been debated for decades. However, recent syntheses suggest a mean elevation of ca. 4,000 m a.s.l. of the central QTP as early as 40 million years ago (Ma; see reviews by Favre et al., 2015; Renner, 2016), with subsequent uplift of high mountain ranges (such as the Himalayas) by early Miocene (Wang et al., 2008; Deng and Ding, 2015). In contrast, the Hengduan Mountains (HM) region is usually considered to have experienced a more recent uplift mainly between Late Miocene and Late Pliocene (Clark et al., 2005; Sun et al., 2011; Wang et al., 2014). The HM, with its outstanding diversity of habitats, is one of the core biodiversity hotspots surrounding the QTP (Favre et al., 2015), harboring ca. 12,000 vascular plants (Li and Li, 1993; Boufford, 2014) with large proportions of alpine elements (Xu et al., 2014). Several spectacular *in situ* radiations have been reported in this region (see Wen et al., 2014). However, numerous phylogenetic studies have inappropriately attributed *in situ* radiations to a scenario of recent QTP uplift (see Renner, 2016 and references therein). The effects of recent HM uplift on evolution and diversification of young lineages were largely ignored, although floristic assembly and diversification rate analyses revealed uplift-driven diversification of plants in the HM (Xing and Ree, 2017).

*Saxifraga* L. is the largest genus in the Saxifragaceae s.str., consisting of 450–500 species (Ebersbach et al., 2017a). Its distribution is concentrated in the mountains of Europe and Asia, with a few species having a circum-polar distribution and some extending to the Andes and Tierra del Fuego (Gao et al., 2015; Tkach et al., 2015; Ebersbach et al., 2017a). The QTP and its flanking mountains, the Himalayas and HM, is a biodiversity center of *Saxifraga*, harboring nearly half the species, mainly represented by the most species-rich section *Ciliatae* Haworth (ca. 175 species, of which more than 150 occur in the QTP and its flanking mountains) (Pan et al., 2001; Ebersbach et al., 2017a). Previous phylogenetic and biogeographic studies on *Saxifaga* revealed the monophyly of sect. *Ciliatae* (Gao et al., 2015; Tkach et al., 2015) and suggested that it diversified *in situ* in the HM region (Ebersbach et al., 2017a), particularly rapidly for subsect. *Hirculoideae* (Ebersbach et al., 2017b). Although *Saxifraga* has been widely employed in phylogeographic studies to reveal patterns and processes of diversification in the arctic and alpine regions (e.g., Abbott and Comes, 2003; Healy and Gillespie, 2004; DeChaine et al., 2013; Gao et al., 2015; Ebersbach et al., 2017a,b), the evolution and diversification of sect. *Ciliatae* are still poorly studied. Tracing genetic divergence between closely related species in sect. *Ciliatae* can give us a better understanding of association between diversification of alpine plants and recent HM uplift as well as Quaternary climatic dynamics. Besides, such an approach can be an effective aid to species delimitation, since many closely related species of sect. *Ciliatae* are not well distinguished based on morphology.

**Figure 1:**
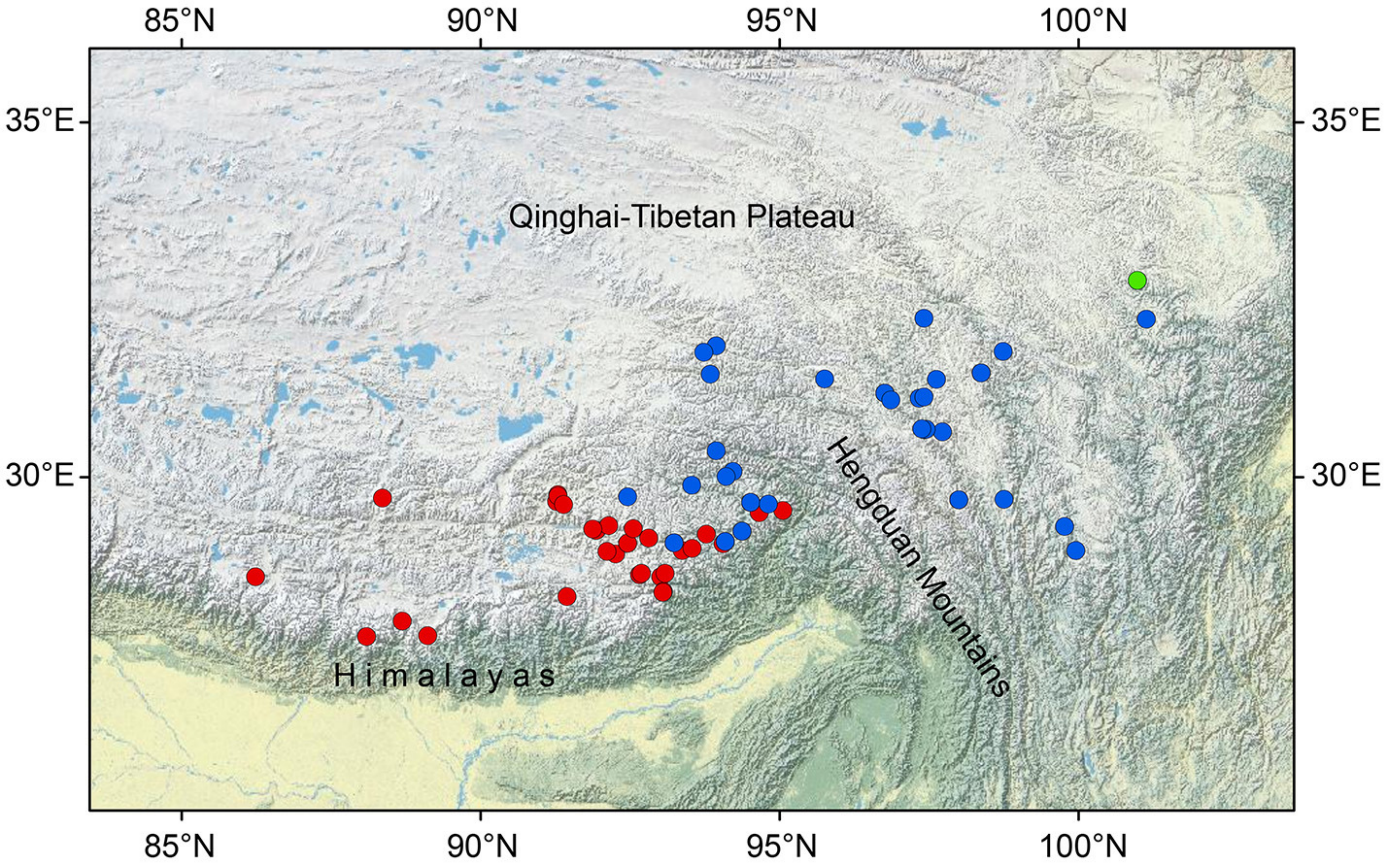
Geographical distribution of *Saxifraga umbellulata* (red spots), *S. pasumensis* (blue spots) and *S. banmaensis* (green spot). Data are based on specimens studied by us in BM, HNWP, KUN, and PE.

The present study focuses on three closely related *Saxifraga* species of unclear taxonomic status: *Saxifraga umbellulata* J. D. Hooker and Thomson, *S. pasumensis* C. Marquand and Airy-Shaw and *S. banmaensis* J-T. Pan. All three taxa belong to sect. *Ciliatae* subsect. *Rosulares* and are characterized by well-defined rosettes of basal leaves and an umbelliform inflorescence of flowers with yellow, pandurate to linear petals. The difference between them is that in *S. umbellulata* the margins of the basal leaves are entire, whereas in *S. pasumensis* and *S. banmaensis* they are cartilaginous and setose-ciliate. The last species is said to differ from *S. pasumensis* by its mucronulate, rather than acute sepals, and linear rather than pandurate petals (Pan et al., 2006). It is only known from the type locality. Taxonomic status and relationships of *S. banmaensis* have not yet been investigated in detail. In addition, based upon the phylogenetic tree of Tkach et al. (2015), *S. umbellulata* and *S. pasumensis* (referred to as *S. umbellulata* var. *pectinata* in Tkach et al., 2015) seem not to be monophyletic, forming a rather unresolved clade. The three taxa share a similar habitat, i.e., alpine rock crevices and cliff faces at elevations of between 3,000 m and 4,600 m a.s.l. Besides, the three species studied here have contrasting geographical distributions. *Saxifraga umbellulata* occurs in the high rolling plateau of southern Tibet and the neighboring Himalayas; *S. pasumensis* occurs mainly in the HM resion; and *S. banmaensis* is known from a single locality, Banma in Qinghai province, at the north-eastern end of the HM system (Figure 1; An et al., 2001; Pan et al., 2006). Populations are typically small and usually isolated from one another.

In this study, we employed three cpDNA fragments (*rbcL, trnL-F, trnS-G*) and nrDNA ITS, of which, *rbcL* and ITS are widely used as DNA barcodes (Hollingsworth et al., 2009), to reveal the population genetic structure and diversification of this group of taxa. In particular, we want to address: (1) population genetic structure of each taxon; (2) the relationship between taxa diversification and geological and climatic events associated with the episodes of uplifting of the HM and Quaternary glaciations; (3) taxonomic relationships among the three taxa.

## Materials and methods

### Taxon delineation

A preliminary morphological study of the type material of *S. banmaensis* (HNWP: *Chen 03031*) plus additional plants from the type locality (HNWP: *Chen 2008022*) showed that some individuals have pandurate-oblong petals with a clawed base rather than linear ones as described by Pan et al. (2006) and that there is a gradation between the two states. Although the diagnostic trait between *S. banmaensis* and *S. pasumensis* (namely mucronate sepals in the former and acute in the latter) seems to be stable, we doubt that the two taxa can be easily distinguished morphologically. In the following analyses, therefore, it has sometimes proved informative to include *S. banmaensis* with *S. pasumensis* as *S. pasumensis* s.l. and distinguish this entity from *S. pasumensis* s.s. (i.e., without *S. banmaensis*). We also note that Pan et al. (2001) regarded *S. pasumensis* as a variety of *S. umbellulata*, i.e., as *S. umbellulata* var. *pectinata* (C. Marquand and Airy-Shaw) J-T. Pan. However, two of Pan's co-authors (R.J. Gornall and H. Ohba) advocated treating *S. pasumensis* as a species separate from *S. umbellulata*, on account of the leaf margin difference (Pan et al., 2001), and this is how it is provisionally referred to here.

### Population sampling strategy

**Figure 2:**
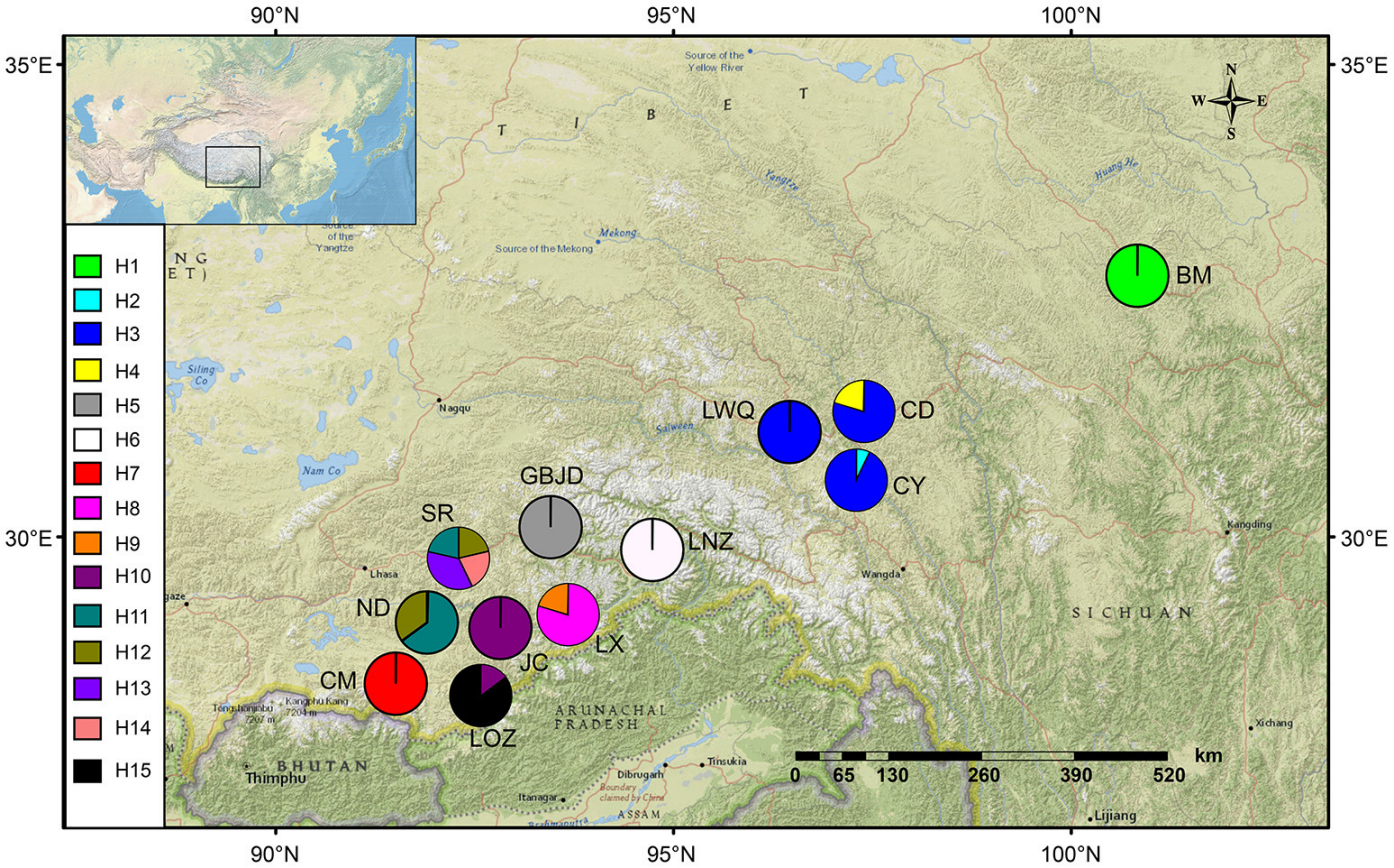
Geographical distribution of sampled populations of *Saxifraga umbellulata* (CM, LX, JC, ND, SR, LOZ), *S. pasumensis* (CY, CD, GBJD, LWQ, LNZ) and *S. banmaensis* (BM). The frequencies of cpDNA haplotypes within each population are shown in the pie charts.

Leaf material of *S. umbellulata, S. pasumensis*, and *S. banmaensis* is extremely difficult to get owing to the relative inaccessibility of their populations. Nevertheless, we sampled all three species from across much of their respective ranges. Taking into account the size of each population, fresh leaves from between 1 and 20 individuals, spaced at least 5 m apart, were sampled. In total, samples from 104 individuals from 12 populations were collected (Table 1, Figure 2). Leaves were dried in silica gel. Voucher specimens of all populations are deposited in the herbarium of Northwest Institute of Plateau Biology (HNWP), Xi'ning, Qinghai, China. *Saxifraga sinomontana* J-T. Pan and Gornall, which is also in sect. *Ciliatae*, was used as outgroup in the phylogenetic analyses.

**Table 1:** Population code (Pop.), sampling location, coordinates, altitude, number of investigated individuals (*n*), cpDNA haplotype and ITS genotype composition of investigated populations of *Saxifraga umbellulata, S. pasumensis* and *S. banmaensis*.

**Pop**.	**Location**	**Coordinates**	**Altitude (m)**	***n***	**Haplotype/genotype composition**	
					**cpDNA**	**ITS**
***Saxifraga umbellulata*** **C. MARQUAND AND AIRY SHAW**						
CM	Cuomei, Tibet	N 28°15′26″ E 91°13′41″	4240	2	H7(2)	G2(1); G3(1)
LX	Langxian, Tibet	N 29°10′12″ E 93°37′05″	3000	5	H8(4); H9(1)	G17(5)
JC	Jiacha, Tibet	N 29°06′59″ E 92°39′14″	3210	1	H10(1)	G19(1)
ND	Naidong, Tibet	N 28°53′22″ E 91°57′13″	4280	20	H11(13); H12(7)	G26(16); G27(4)
SR	Sangri, Tibet	N 29°15′23″ E 92°23′50″	3040	14	H11(3); H12(3); H13(5); H14(3)	G19(1); G20(4); G22(2); G23(3); G24(3); G28(1)
LOZ	Longzi, Tibet	N 28°19′06″ E 92°53′35″	3050	20	H10(3); H15(17)	G18(1); G21(1); G25(1); G29(1); G30(5); G31(6); G32(2); G33(2); G34(1)
***S. pasumensis*** **J. D. HOOKER AND THOMSON**						
CY	Chaya, Tibet	N 30°41′11″ E 97°15′42″	4090	14	H2(1); H3(13)	G4(4); G5(8); G6(2)
CD	Changdu, Tibet	N 31°09′20″ E 97°15′27″	4300	5	H3(4); H4(1)	G6(4); G8(1)
GBJD	Gongbujiangda, Tibet	N 29°53′03″ E 93°22′14″	3360	3	H5(3)	G10(2); G11(1)
LWQ	Leiwuqi, Tibet	N 31°06′40″ E 96°42′14″	3770	8	H3(8)	G5(4); G7(2); G9(2)
LNZ	Linzhi, Tibet	N 29°36′46″ E 94°39′19″	4540	8	H6(8)	G12(1); G13(1); G14(1); G15(3); G16(2)
***S. banmaensis*** **J-T. PAN**						
BM	Banma, Qinghai	N 32°48′46″ E 100°49′28″	3460	4	H1(4)	G1(3); G3(1)

### DNA extraction, PCR amplification and sequencing

Total genomic DNA was extracted from silica-dried leaves using the modified CTAB method of Doyle and Doyle (1987). For the polymerase chain reactions (PCR), primers “*c*” and “*f*” for *trnL*-*trnF* (Taberlet et al., 1991), “trnS^GCU^” and “trnG^UCC^” for *trn*S-*trn*G (Hamilton, 1999) and “ITS1a” and “ITS4” for ITS (White et al., 1990) were used for amplification. PCR mixtures and amplification programs followed Gao et al. (2012, 2015). For the *rbcL* fragment, PCR was performed with primers “1F” and “724R” (Fay et al., 1997), in a 50 μL volume, containing 1.0 μL genomic DNA (approximately 30 ng), 5.0 μL of 10 × PCR buffer (with Mg^2+^), 2.0 μL of 10 mM dNTPs, 1.0 μL of 5 pM of each primer and 0.3 μL (1.5 units) of Taq polymerase. The amplification profile was: 6 min at 94°C, then 35 cycles of 50 s at 94°C, 50 s at 52.5°C, 1 min at 72°C, followed by a final extension of 7 min at 72°C. The PCR products were purified using a CASpure PCR Purification Kit (CASarray, Shanghai, China), and then sequenced in both directions with the primers used for amplification on an ABI PRISM 3730xl analyser.

### Haplotype isolation

Parallel chromatograms derived from bi-directional sequencing were contrasted for accuracy by visual inspection using Chromas ver. 2.6.4 (available at http://www.technelysium.com.au). DNA sequences were aligned in MEGA ver. 7.0.26 (Kumar et al., 2016) with minor subsequent adjustments. The ragged tails of the alignments were trimmed to ensure a uniform ending. All sequences were assigned to different haplotypes (for cpDNA sequences) or genotypes (for ITS sequences) using DnaSP ver. 5.10 (Librado and Rozas, 2009). All newly generated sequences in this study, including that of *S. sinomontana* (used as outgroup), have been deposited in GenBank (accession numbers MF197564-MF197628). The three cpDNA fragments (*rbcL, trnL-F*, and *trnS-G*) were then concatenated into a single matrix for subsequent analyses.

### Phylogenetic analysis

Phylogenetic relationships of the chloroplast haplotypes and nuclear ITS genotypes were reconstructed by means of Maximum Parsimony (MP), Maximum Likelihood (ML) and Bayesian Inference (BI). The MP analyses were conducted in PAUP 4.10b (Swofford, 2002) with gaps treated as missing characters. A heuristic search with 100 random-taxon-addition replicates was performed with tree bisection-reconnection (TBR) branch swapping, and MulTrees. Estimates of bootstrap support (BS) were calculated using 1,000 replicates of a heuristic search with simple addition, with TBR and MULPARS in effect. For ML and BI analyses, best-fit substitution models were chosen by the Akaike Information Criteria (AIC) using jModelTest ver. 2.1.4 (Darriba et al., 2012). Models of GTR+I and SYM+G were selected for the cpDNA and ITS datasets, respectively. The ML analyses were conducted using RAxML ver. 8.1.21 (Stamatakis, 2014) implemented in raxmlGUI ver. 1.5b2 (Silvestro and Michalak, 2012) with a selection of ML + rapid bootstrap and support assessment using 1,000 rapid bootstraps. The BI analyses were performed using MrBayes ver. 3.2.6 (Ronquist and Huelsenbeck, 2003; Ronquist et al., 2012). The models of evolution used were the same as those used for the ML analyses. Two simultaneous Markov Chain Monte Carlo (MCMC) analyses were run for ten million generations, saving one tree every 1,000 generations with the first quarter of the trees discarded as burn-in.

### Population genetic structure analysis

Spatial analysis of molecular variance (SAMOVA) for cpDNA and ITS datasets were carried out using SAMOVA ver. 2.0 (Dupanloup et al., 2002) to determine population groups that are geographically homogenous and maximally differentiated from each other. *K*-values were increased from 2 to 5. Average within-population diversity (*H*_*S*_), total gene diversity (*H*_*T*_) and differentiation for unordered and ordered alleles, *G*_*ST*_ and *N*_*ST*_, were estimated for the overall genepool and for populations of *S. umbellulata* and *S. pasumensis* (s.l. and s.s.), respectively, using PERMUT (Pons and Petit, 1996; available at http://www.pierroton.inra.fr/genetics/labo/Software/PermutCpSSR). As an indicator of phylogeographic structure, a comparison was made between *G*_*ST*_ and *N*_*ST*_ using a permutation test with 1,000 permutations. Since PERMUT software requires at least three individuals per population, populations CM (two individuals) and JC (one individual) were discarded during PERMUT analysis. Analysis of molecular variance (AMOVA) was performed using ARLEQUIN ver. 3.5.2 (Excoffier and Lischer, 2010) to partition genetic variation into different levels, with the statistical significance determined by 1,000 permutations. To further measure DNA divergence between putative species, we then divided all populations into four subsets: (1) population BM, which represents *S. banmaensis*; (2) *S. pasumensis* s.s., comprising populations CY, CD, GBJD, LWQ, LNZ; ((3) *S. umbellulata*, comprising populations CM, LX, JC, ND, SR, LOZ; and (4) *S. pasumensis* s.l., containing populations BM, CY, CD, GBJD, LWQ, LNZ. Pairwise comparisons of *F*_*ST*_ between *S. banmaensis, S. pasumensis* s.s. and *S. umbellulata*, and between *S. pasumensis* s.l. and *S. umbellulata* were calculated using ARLEQUIN ver. 3.5.2 (Excoffier and Lischer, 2010), and the significance was tested using 1,000 permutations.

### Population demographic analysis

To infer demographic processes, mismatch distribution analyses based on the cpDNA dataset were conducted for the overall genepool and for populations of *S. umbellulata* and *S. pasumensis* (s.l. and s.s.), respectively. The shape of the frequency of pairwise differences is expected to be multimodal in samples drawn from populations at demographic equilibrium. However, it is usually unimodal in populations having experienced a recent demographic or range expansion (Slatkin and Hudson, 1991; Rogers and Harpending, 1992; Harpending et al., 1998; Ray et al., 2003; Excoffier, 2004). The sum of squared deviations (SSD) and Harpending's raggedness index (HRI, Harpending, 1994) between observed and expected mismatch distributions were used as test statistics. One thousand parametric bootstrap replicates were used to generate an expected distribution under a model of sudden demographic expansion (Excoffier and Lischer, 2010). Neutrality tests using Tajima's *D* (Tajima, 1989) and Fu's *F*_*S*_ (Fu, 1997) were conducted based on the cpDNA dataset to detect departures from population equilibrium. All of these demographic tests were conducted using ARLEQUIN ver. 3.5.2 (Excoffier and Lischer, 2010).

### Divergence time estimation

The ITS dataset was employed to estimate divergence times between recovered lineages using BEAST ver. 1.8.4 (Drummond et al., 2012). Thirty-nine outgroups and three fossil calibrations were employed to get a more reliable age estimation. The 39 outgroups include: (1) all six remaining subsections of sect. *Ciliatae* that were identified by Gao et al. (2015); (2) all 12 remaining sections of *Saxifraga* as identified by Tkach et al. (2015); (3) eleven other genera of Saxifragaceae; 4) two other families of Saxifragales (Table S4). We followed Ebersbach et al. (2017a) in their use of *Ribes* and *Itea* fossils to set *Ribes* stem node and MRCA of *Itea* and *Pterostemon* to be 48.9 and 49 Ma, respectively. Log-normal prior distributions were used with a mean of 1.5 and a standard deviation of 1.0. We followed Zhu et al. (2013) in their use of *Divisestylus* to calibrate the stem age of the *Itea*- *Pterostemon* using a mean age of 91.4 Ma with the standard error of 1.0.

The BEAUti interface was used to create an input file for BEAST, to which GTR+I+G nucleotide substitution model, the Yule tree prior and uncorrelated log-normal clock model were applied. The Bayesian Markov Chain Monte Carlo simulation was run for 50 million generations with a sample frequency of 5,000, and the first 25% generations were discarded as burn-in. Two independent analyses were conducted and combined by LogCombiner ver. 1.8.4 (available at http://beast.bio.ed.ac.uk/LogCombiner) with a 25% burn-in for each run. The maximum clade credibility tree was summarized in TreeAnnotator ver. 1.8.4 (available at http://beast.bio.ed.ac.uk/TreeAnnotator) with the posterior probability limit set to 0.5 and summarizing mean node heights. The final calibrated chronogram and their 95% highest posterior density (95% HPD) were visualized and edited using FigTree ver. 1.4.3 (available at http://tree.bio.ed.ac.uk).

## Results

### Haplotype/genotype phylogenetics and distribution

**Figure 3:**
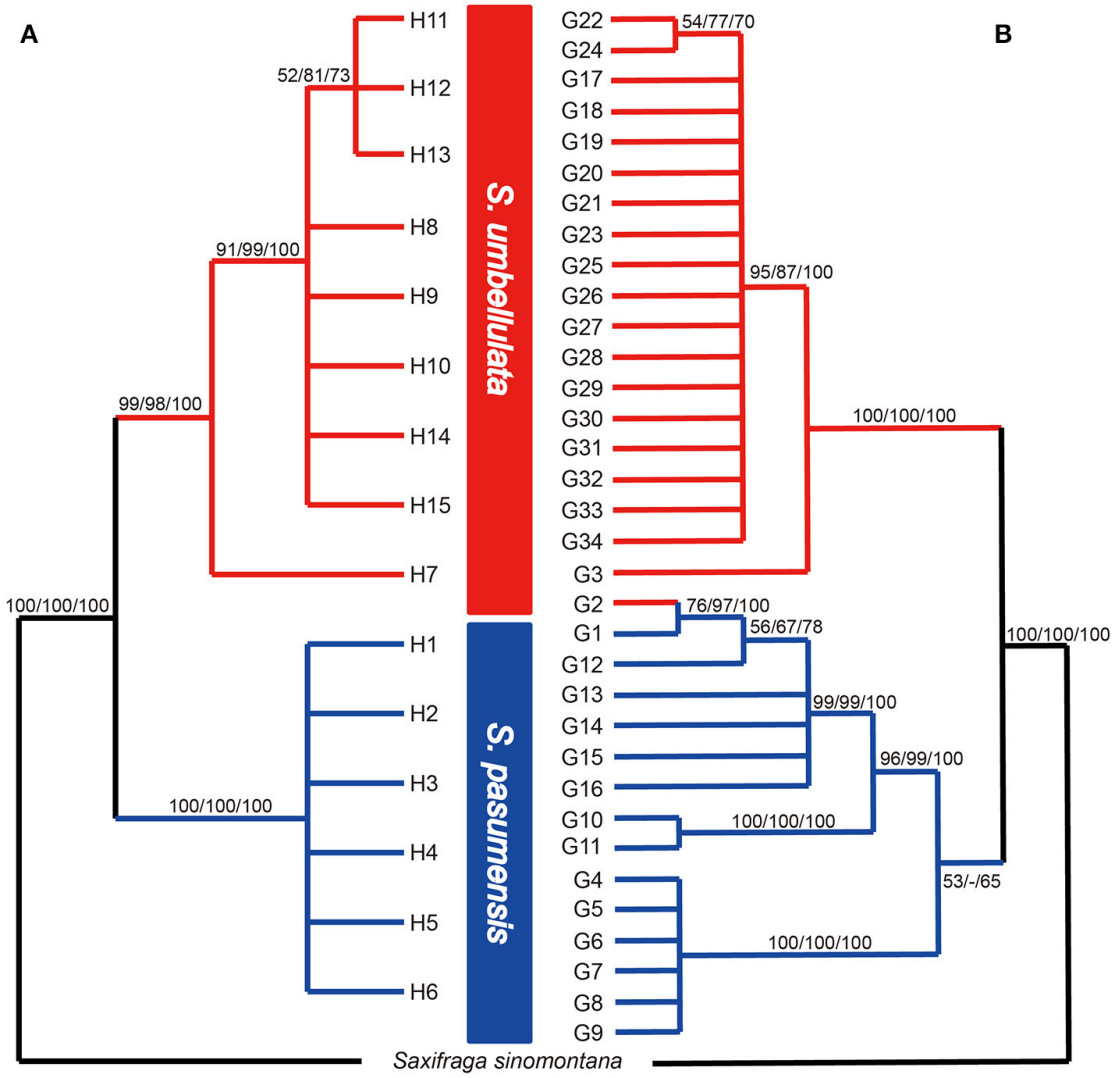
Fifty percent strict consensus trees based on cpDNA haplotypes **(A)** and ITS genotypes **(B)** of *Saxifraga pasumensis* s.l. and *S. umbellulata*. Numbers on the branches are bootstrap values from maximum parsimony (left) and maximum likelihood (middle) analyses and posterior probabilities (right) from Bayesian inference.

Based on the concatenated cpDNA sequences (*rbcL, trnL-F, trnS-G*), 15 haplotypes (H1-H15) were identified among the 104 individuals of *S. pasumensis, S. banmaensis* and *S. umbellulata*. Sequence lengths of these haplotypes varied from 1987 to 2,115 bp with an alignment length of 2,134 bp. Variable sites among the 15 haplotypes are shown in Table S1. No haplotypes were shared by populations from different species, and 11 of the 15 haplotypes were private, i.e., confined to single populations. Of those that were shared, haplotype H3 occurred in three geographically close populations (CY, CD, LWQ) of *S. pasumensis* (Table 1, Figure 2). In *S. umbellulata*, haplotype H10 was shared by populations JC and LOZ, and haplotypes H11 and H12 were shared by populations ND and SR. Regarding the phylogenetic relationships of the haplotypes, the topologies of the trees reconstructed by MP, ML, and BI methods were congruent for the major clades. That is, the 15 haplotypes were clustered into two well supported clades, comprising haplotypes from populations of *S. pasumensis* plus *S. banmaensis* (100% BS MP, 100% BS ML, 100% PP BI) and those of *S. umbellulata* (99% BS MP, 98% BS ML, 100% PP BI), respectively (Figure 3A). Relationships of haplotypes within each clade, however, were largely unresolved.

A total of 71 single nucleotide polymorphisms was detected among the 104 ITS sequences of *S. pasumensis, S. banmaensis*, and *S. umbellulata*. This allowed the recognition of 34 genotypes (G1-G34) (Table S2). Phylogenetic relationships of these genotypes generated by means of MP, ML and BI revealed two major clades, one comprised of *S. pasumensis* plus *S. banmaensis* and the other of *S. umbellulata* (Figure 3B). This ITS tree topology was largely congruent with that generated from cpDNA haplotypes, but with some minor conflicts as follows. (1) Genotype G3 was shared by the two most distant populations, BM (*S. banmaensis*) and CM (*S. umbellulata*) (Table 1). This shared genotype clustered with those of *S. umbellulata* (Figure 3B). (2) Genotype G2 was confined to population CM of *S. umbellulata* (Table 1), but clustered within the *S. pasumensis* clade. (3) The *S. pasumensis* plus *S. banmaensis* clade recovered by ITS genotypes was weakly supported compared to that on the cpDNA tree topology (53% BS MP, <50% BS ML, 65% PP BI). (4) Relationships between genotypes within the *S. pasumensis* plus *S. banmaensis* clade were better resolved than those of *S. umbellulata* (Figure 3B).

### Population genetic structure and demographic history

Spatial analysis of molecular variance (SAMOVA) for cpDNA and ITS datasets with *K* = 2 resulted in groups corresponding exactly to the two species, *S. umbellulata* and *S. pasumensis* s.l., as identified based on morphology (Table S3). However, the *F*_*CT*_ value increased steadily when *K* increased from 2 to 5 based on both cpDNA and ITS datasets. In accordance with morphological traits and phylogenetic clustering, we therefore chose *K* = 2 as the most appropriate division of the overall population. The cpDNA and ITS datasets revealed high total gene diversity (*H*_*T*_) both for the overall populations and for each of the two species, *S. umbellulata* and *S. pasumensis* (s.l. and s.s.) (Table 2). However, values of average within-population diversity (*H*_*S*_) revealed by the ITS dataset were higher than those revealed by the cpDNA dataset both for individual species and overall, which may due to a large number of hybridization sites of ITS sequences (Table S2) that over-estimate average gene diversity within populations. For both cpDNA and ITS datasets, an estimation of significantly larger *N*_*ST*_ than *G*_*ST*_ values across all populations suggests that genetic variation is geographically structured across the entire distribution range. This is also the case for each species except in the cpDNA dataset for *S. pasumensis* s.s. (Table 2). A two-level AMOVA based on both cpDNA and ITS datasets for populations of *S. pasumensis* s.l. and *S. umbellulata* revealed that total genetic variation was largely attributed to the differences among populations (*S. pasumensis* s.l.: 95.29% of cpDNA, 87.94% of ITS; *S. umbellulata*: 78.32% of cpDNA, 75.47% of ITS). Further analysis by hierarchical AMOVA showed that variation between *S. pasumensis* s.l. and *S. umbellulata* amounted to 92.31% of the total for cpDNA, but 69.78% for ITS (Table 3).

**Table 2:** Estimates of average gene diversity within populations (*H*_*S*_), total gene diversity (*H*_*T*_), inter-population differentiation (*G*_*ST*_) and number of substitution types (*N*_*ST*_) for cpDNA haplotypes and ITS genotypes of *Saxifraga umbellulata* and *S. pasumensis* (s.l. and s.s.).

	**cpDNA**				**ITS**			
**Groups**	***H_*S*_***	***H_*T*_***	***G_*ST*_***	***N_*ST*_***	***H_*S*_***	***H_*T*_***	***G_*ST*_***	***N_*ST*_***
*S. umbellulata*	0.485	0.964	0.497	0.808^**^	0.513	1.000	0.487	0.650^**^
*S. pasumensis* s.l.	0.090	0.835	0.892	0.945^*^	0.626	0.973	0.357	0.796^**^
*S. pasumensis* s.s.	0.109	0.753	0.856	0.937^*ns*^	0.651	0.960	0.322	0.923^**^
Over all populations	0.248	0.940	0.736	0.933^**^	0.581	0.991	0.414	0.873^**^

ns, not significant;

* P < 0.05;

** *P < 0.01*.

**Table 3:** Analysis of molecular variance (AMOVA) of cpDNA haplotypes and ITS genotypes for populations of *Saxifraga umbellulata* and *S pasumensis* s.l.

		**cpDNA**				**ITS**			
**Source of variation**	***df***	***SS***	***VC***	***PV* (%)**	***F_*ST*_***	***SS***	***VC***	***PV* (%)**	***F_*ST*_***
***S. umbellulata***									
Among populations	5	16.899	0.361	78.32		63.941	1.358	75.47	
Within populations	56	5.592	0.100	21.68		24.724	0.442	24.53	
Total	61	22.491	0.461		0.7832^*^	88.665	1.800		0.7547^*^
***S. pasumensis*** **s.l**.									
Among populations	5	70.110	2.103	95.29		230.013	6.809	87.94	
Within populations	36	3.740	0.104	4.71		33.610	0.934	12.06	
Total	41	73.849	2.207		0.9529^*^	263.623	7.743		0.8794^*^
***S. umbellulata*****, VS**. ***S. pasumensis*** **s.l**.									
Among species	1	749.502	14.696	92.31		541.366	9.921	69.78	
Among populations	10	89.114	1.121	7.04		293.954	3.662	25.76	
Within populations	92	9.475	0.103	0.65		58.334	0.634	4.46	
Total	103	848.090	15.920		0.9935^*^	893.625	14.217		0.9554^*^

df, degrees of freedom; SS, sum of squares; VC, variance components; PV, percentage of variation; F_ST_, fixation index;

* *P < 0.01*.

To further estimate genetic divergence between putative species, we then divided all populations into four combinations (see Materials and Methods). Pairwise *F*_*ST*_ values between the four combinations are shown in Table 4. Both cpDNA and ITS datasets revealed high levels of divergence between *S. pasumensis* combinations (BM, *S. pasumensis* s.s. and s.l.) and *S. umbellulata*. However, pairwise *F*_*ST*_ value between population BM, which was described as *S. banmaensis* (Pan et al., 2006), and the remaining populations of *S. pasumensis* was relatively low as revealed by ITS dataset (0.4525), even negative as by cpDNA dataset (−0.2919) (Table 4).

**Table 4:** Pairwise comparisons of *F*_*ST*_ between populations of *Saxifraga banmaensis, S. pasumensis* s.s. and *S. umbellulata*, and between populations of *S. pasumensis* s.l. and *S. umbellulata*, based on cpDNA (lower triangle) and ITS (upper triangle) datasets, respectively.

	***S. umbellulata***	***S. pasumensis* s.l**.	***S. pasumensis* s.s**.	***S. banmaensis***
*S. umbellulata*		0.7567^**^	0.7942^**^	0.8594^**^
*S. pasumensis* s.l.	0.9393^**^		–	–
*S. pasumensis* s.s.	0.9364^**^	–		0.4525^*^
*S. banmaensis*	0.9757^**^	–	−0.2919^ns^	

ns, not significant;

* P < 0.01;

** *P < 0.001*.

**Figure 4:**
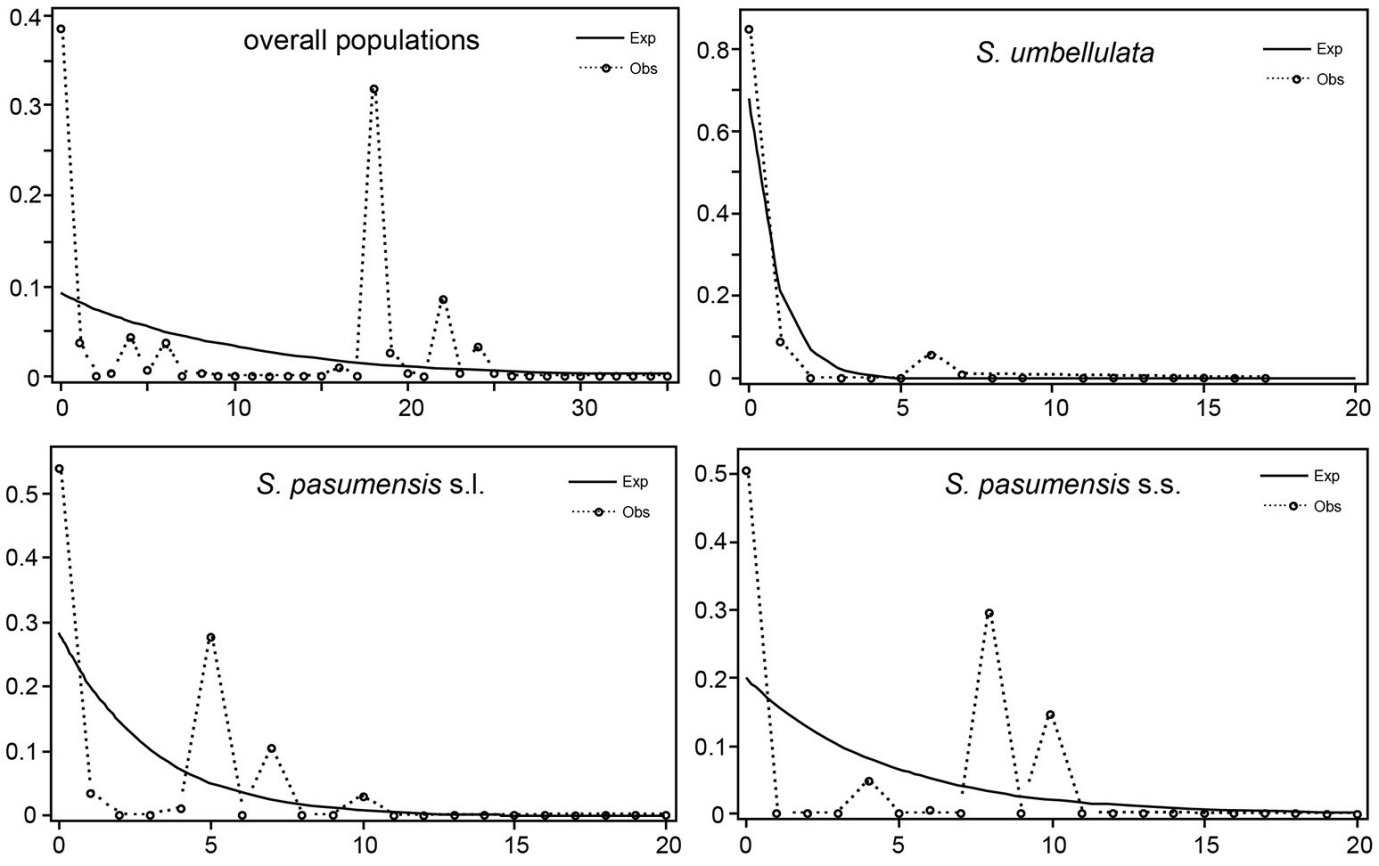
Mismatch distribution of *Saxifraga pasumensis* (s.l. and s.s) and *S. umbellulata* in the overall populations and in each species based on the cpDNA dataset.

Neutrality tests of Tajima's *D* and Fu's *F*_*S*_ based on cpDNA data showed positive values for the whole gene pool (Table 5), suggesting a rejection of recent expansion across the distribution range of the three species. This was supported by mismatch distribution analysis, in which multimodal was drawn from the overall populations, indicating a demographic equilibrium (Figure 4). The same picture was also found for populations of *S. pasumensis* (s.s. and s.l). Non-significant negative value, or positive values of Tajima's *D* and Fu's *F*_*S*_ and multimodal of mismatch distributions, all suggest a demographic equilibrium of *S. pasumensis* (s.s. and s.l.) (Table 5, Figure 4). However, recent expansion could not be rejected for *S. umbellulata* populations as suggested by a significant negative value of Tajima's *D* and unimodal of mismatch distributions (Table 5, Figure 4).

**Table 5:** Neutrality tests (Tajima's D, Fu's F) and mismatch distribution analysis for the combined populations and the two species of *Saxifraga umbellulata* and *S. pasumensis* (s.l. and s.s.) based on the cpDNA dataset.

	**Tajima's** ***D*****-test**		**Fu's** ***Fs*****-test**		**Mismatch distribution**				
**Populations**	***D***	***P***	***Fs***	***P***	***Tau***	***HRI***	***P***	***SSD***	***P***
Overall	2.638	0.996	21.225	0.997	0.000	0.176	0.05	0.145	0.381
*S. umbellulata*	−1.732	0.014	3.401	0.937	3.000	0.586	0.675	0.005	0.246
*S. pasumensis* s.l.	−0.070	0.509	3.598	0.927	0.000	0.386	0.967	0.328	0.000
*S. pasumensis* s.s.	0.674	0.802	5.840	0.971	0.000	0.420	0.938	0.384	0.000

*SSD, sum of squared deviation under expansion model; HRI, Harpending's raggedness index*.

### Estimation of divergence times

**Figure 5:**
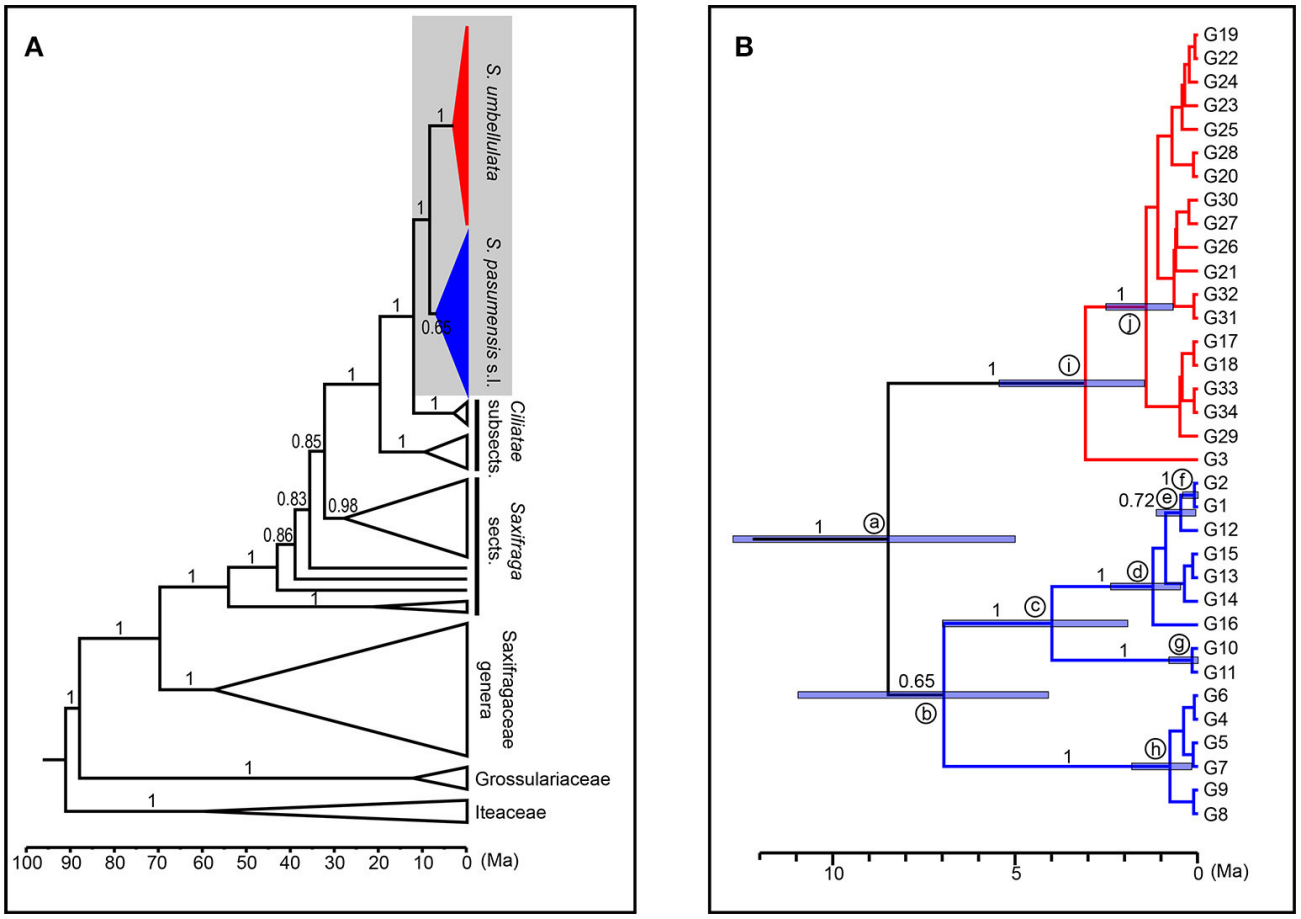
Maximum clade credibility tree and divergence time estimation based on ITS genotypes of *Saxifraga pasumensis* s.l. and *S. umbellulata*. **(A)** Profile of time estimation results including all outgroups. **(B)** Enlargement of the shadowed area, which represents ITS genotypes of *S. pasumensis* s.l. and *S. umbellulata*. Blue bars represent 95% highest posterior density. Numbers on the branches are values of posterior probabilities.

We used ITS genotypes to estimate divergence times between discovered clades, since the ITS dataset better resolved genotypic relationships within species. Thirty-nine outgroups and three fossil calibrations were employed to get a more accurate age estimation. Consistent and robust nodes of ITS genotypes of *S. pasumensis, S. banmaensis*, and *S. umbellulata* are marked on Figure 5 and the results are outlined in Table 6. The stem of Saxifragaceae was estimated to 88.09 Ma (95% HPD = 78.22–92.82 Ma). Stem *Saxifraga* was dated to 69.83 Ma (95% HPD = 58.14–80.29 Ma), with crown *Saxifraga* to 54.22 Ma (95% HPD = 42.99–66.37 Ma). The stem and crown of section *Ciliatae* were estimated to 32.40 Ma (95% HPD = 24.29–41.24 Ma) and 19.79 Ma (95% HPD = 13.41–27.92 Ma), respectively. The divergence of *S. pasumensis* s.l. and *S. umbellulata* was dated to 8.50 Ma (node a, 95% HPD = 5.01–12.72 Ma), coinciding with the orogenic events of the HM between Late Miocene and Late Pliocene (Favre et al., 2015). Infra-specific divergence in *S. pasumensis* s.l. was estimated to start at 6.97 Ma (node b, 95% HPD = 4.10–10.97 Ma), earlier than in *S. umbellulata* where divergence began at 3.09 Ma (node i, 95% HPD = 1.47–5.46 Ma). Nearly all *S. umbellulata* ITS genotypes had diverged by 1.42 Ma (node j, 95% HPD = 0.68–2.53 Ma).

**Table 6:** Estimation of divergence times for consistent and robust nodes of ITS genotypes of *Saxifraga umbellulata* and *S. pasumensis* s.l.

**Node**	**Mean age (Ma)**	**95% HPD**
Saxifragaceae stem	88.09	78.22–92.82
*Saxifraga* stem	69.83	58.14–80.29
*Saxifraga* crown	54.22	42.99–66.37
sect. *Ciliatae* stem	32.40	24.29–41.24
sect. *Ciliatae* crown	19.79	13.41–27.92
a	8.50	5.01–12.72
b	6.97	4.10–10.97
c	4.01	1.93–7.01
d	1.24	0.48–2.40
e	0.47	0.06–1.14
f	0.10	0.00–0.42
g	0.16	0.00–0.80
h	0.77	0.17–1.82
i	3.09	1.47–5.46
j	1.42	0.68–2.53

*Node numbers (a–j) are as those indicated in Figure 5B. HPD, highest posterior density*.

## Discussion

### Divergence and evolutionary history of *S. pasumensis* s.l. and *S. umbellulata*

Molecular clock analyses are commonly employed to estimate the temporal framework of diversification of studied lineages, which is then used to trace the association with geologic/climatic events. Factors such as taxa-sampling density and fossil calibration strategy can drastically bias age estimates (Linder et al., 2005; Sauquet et al., 2012; Favre et al., 2015; Ebersbach et al., 2017b). In this study, we employed 39 outgroups and three fossil calibrations to get a more accurate divergence time estimation of ITS genotypes of *S. pasumensis, S. banmaensis*, and *S. umbellulata*. Although our taxon coverage is still limited, it represents all the 13 sections of *Saxifaga* (Tkach et al., 2015) and the seven subsections of sect. *Ciliatae* (Gao et al., 2015). Our divergence time estimates for stems of Saxifragaceae (mean age of 88 Ma) and *Saxifraga* (mean age of 70 Ma) are comparable to those of median ages (84 and 74 Ma for stems of Saxifragaceae and *Saxifraga*, respectively) estimated by Ebersbach et al. (2017a), who employed the most extensive coverage to date. Nevertheless, divergence time estimates in this study should be treated with caution, since limited taxon coverage and low number of fossil calibrations might bias divergence time estimation toward younger ages as detected for *Saxifraga* crown (mean age of 54 Ma) as well as stem and crown of sect. *Ciliatae* (mean age of 32 and 20 Ma, respectively) in this study, compared to those of median ages estimated by Ebersbach et al. (2017a) (*Saxifraga* crown: 62 Ma; sect. *Ciliatae* stem: 41 Ma; sect. *Ciliatae* crown: 26 Ma). However, in this study, molecular clock analysis based on ITS genotypes indicate that the estimated divergence time of *S. pasumensis* s.l. and *S. umbellulata* was 8.50 Ma (95% HPD = 5.01–12.72 Ma), coinciding with orogeny of the HM. In comparison with the rest of the QTP, the uplift of the HM is generally believed to have been more recent, occurring mainly between Late Miocene and Pliocene (Favre et al., 2015). The uplift of the HM probably caused habitat diversification (Favre et al., 2015; Ebersbach et al., 2017b), which might have triggered *in situ* radiations in many plant lineages in the region (Xing and Ree, 2017). Various mechanisms have been proposed to explain plant radiations and diversification in the QTP and its flanking mountains (Wen et al., 2014). Among these mechanisms, allopatric divergence involving vicariance processes associated with orogenic events have been proposed as the principal mechanism of species diversification of both flora and fauna (e.g., Yang et al., 2009; Xu et al., 2010). Considering the highly isolated populations of the studied species, divergence of *S. pasumensis* s.l. and *S. umbellulata* seems to be caused by geographic isolation associated with recent HM uplift events. The fact that the cpDNA dataset also indicates the presence of the same two strongly supported clades, corresponding to *S. umbellulata* and *S. pasumensis* s.l., together with the fact that no cpDNA haplotypes are shared between them, suggests that no subsequent seed-mediated gene flow has occurred between the two species since their divergence.

A word of caution needs to be injected here because there may be other players in this story. In particular, we do not yet know to what extent, if any, two other putatively closely related species, namely *S. muricola* C. Marquand and Airy Shaw and *S. lhasana* H. Smith, may be involved. Furthermore, whether polyploidization played a role in the diversification of the three studied species is still unclear, since chromosome counts are not yet available. Further information on ploidy level would be required to get a better picture of species diversification for *S. pasumensis, S. banmaensis*, and *S. umbellulata*.

Our results might bear the signature of hybridization, as suggested by minor inconsistencies between phylogenies based on nuclear and plastid data. The *S. umbellulata* ITS genotype G3 (in population CM) was also found in *S. banmaensis* (population BM), the two most distant populations in the study; and the *S. pasumensis* ITS genotype G2 was found in the *S. umbellulata* population CM. Although the confusion of genotypic distribution may rise from hybridization sites as detected in many identified genotypes, there might be other reasons. Incongruence between phylogenies of plastid and nuclear DNA is generally explained by convergent evolution, hybridization/introgression or incomplete lineage sorting (ancestral polymorphism) (Rieseberg and Wendel, 1993). In the present case, given the non-coding regions used in this study, convergent evolution is unlikely to be responsible for the incongruence (Yang et al., 2012). Hybridization may be a possibility, and it has been suggested that it played an important role in the rapid diversification of several QTP plant groups, e.g., *Meconopsis* (Yang et al., 2012) and *Rhododendron* (Zha et al., 2010). However, in the present case we would have expected any hybrid signature to be detected in the zone of contact between the two lineages (Wang et al., 2001; Yang et al., 2012), but the two populations involved, BM and CM, are the furthest apart in the study, separated by some 1,200 km, rendering hybridization an unlikely explanation. Nevertheless, we cannot completely reject the possibility of long distance pollen-mediated gene flow (Li et al., 2016), especially if it has involved intervening populations that were not sampled by us. Molecular clock analysis of the ITS dataset suggests that the two genotypes, G2 and G3, have only relatively recently diverged from their nearest relatives: 0.10 Ma (node f, 95% HPD = 0–0.42 Ma) and 3.09 Ma (node i, 95% HPD = 1.47–5.46 Ma), respectively. Given their more recent divergence, it is quite likely that incomplete lineage sorting, which results in the stochastic fixation of ancestral polymorphisms in descendant populations, could be the explanation for the incongruence between ITS and cpDNA phylogenies in this study.

The two clades, *S. pasumensis* s.l. and *S. umbellulata*, may have experienced different evolutionary histories following their divergence. *Saxifraga pasumensis* s.l. occupies mainly the HM region, while *S. umbellulata* is distributed chiefly in southern Tibet. Although the two taxa are largely allopatric, there is a zone of contact (Figure 1). Actually, populations of *S. pasumensis* s.l. employed in this study can be divided into three well isolated geographical partitions: BM (*S. banmaensis*), CD-CY-LWQ and LNZ-GBJD. Molecular clock analysis based on the ITS dataset suggests that the divergence of main lineages in *S. pasumensis* was between 6.97 Ma (node b, 95% HPD = 4.10–10.97 Ma) and 4.01 Ma (node c, 95% HPD = 1.93–7.01 Ma). This coincides with the rapid and recent uplift of HM which was considered to start at Late Miocene and reach its peak elevation shortly before Late Pliocene (Mulch and Chamberlain, 2006; Sun et al., 2011; Favre et al., 2015), suggesting the ongoing contribution of vicariance processes to the infraspecific differentiation of *S. pasumensis* s.l. In fact, the HM is one of the most rugged regions of the world, containing several mountain ranges and deep river valleys (e.g., the Yangzi, the Mekong and the Salween valleys). The physiographic complexity of HM seems to be a predominant factor triggering rapid *in situ* radiations via allopatric speciation (Hughes and Atchison, 2015; Hughes et al., 2015). Neutrality tests and mismatch distribution analysis of populations of *S. pasumensis* and *S. banmaensis* based on the cpDNA dataset suggest that there has been no recent expansion across their distribution range. Thus, vicariance processes associated with the HM uplifts may have been an important promoter of infraspecific divergence in *S. pasumensis* s.l., as has been reported in some other QTP plant species, such as *Hippophae tibetana* (Wang et al., 2010). This is not the picture, however, in *S. umbellulata*, whose distribution range is largely in southern Tibet where topological diversity is lower. Results of neutrality tests and mismatch distribution based on the cpDNA dataset in this species show that the possibility of recent expansion across its distribution range cannot be rejected. Molecular clock analysis of the ITS dataset estimated that nearly all ITS genotypes of *S. umbellulata* have divergence times less than 1.42 Ma (node j, 95% HPD = 0.68–1.53 Ma), which coincides with the four extensive glaciations on the QTP starting at 1.17 Ma (Zheng et al., 2002). It is quite likely that the distribution of *S. umbellulata* expanded before the glaciations and from one or two centers, such as populations SR and LOZ, which harbor high haplotype and/or genotype diversity. Subsequent glacial and interglacial episodes may have fragmented the distribution range of *S. umbellulata* into small, isolated populations, resulting in allopatric infraspecific differentiation. Such a scenario has been suggested in many phylogeographic studies of QTP plants, e.g., *Rhodiola chrysanthemifolia* (Gao et al., 2016), *Rhodiola alsia* (Gao et al., 2012), *Juniperus tibetica* complex (Opgenoorth et al., 2010), *Aconitum gymnandrum* (Wang et al., 2009).

Range contractions and associated population fragmentation could reduce intra-population diversity owing to genetic bottlenecks and drift, but correspondingly increase inter-population differentiation owing to reduced gene flow among populations (Young et al., 1996; Lienert and Fischer, 2003; Arenas et al., 2012; Mona et al., 2014). Small isolated populations might be more vulnerable to environmental changes and human activities. Our cpDNA results reveal that average within-population gene diversity of *S. pasumensis* (*H*_*S*_ = 0.090 for *S. pasumensis* s.l.; *H*_*S*_ = 0.109 for *S. pasumensis* s.s.) is lower than that of *S. umbellulata* (*H*_*S*_ = 0.521) and indeed other herbaceous species in this region, e.g., *Pedicularis longiflora, H*_*S*_ = 0.332, (Yang et al., 2008); *Aconitum gymnandrum, H*_*S*_ = 0.207 (Wang et al., 2009); *Rhodiola alsia, H*_*S*_ = 0.571 (Gao et al., 2012); *Rhodiola chrysanthemifolia, H*_*S*_ = 0.411 (Gao et al., 2016). A two-level AMOVA analyses based on cpDNA and ITS datasets revealed higher among-population differentiation in *S. pasumensis* s.l. than in *S. umbellulata*. Although loss of genetic variation due to bottlenecks during contraction and fragmentation and subsequent genetic drift could be responsible for the extremely low level of average within-population gene diversity of *S. pasumensis* s.l., it is possible that harvesting by people might also be a contributory cause. *Saxifraga pasumensis* has been used in traditional folk medicine to treat hepatitis and cholecystitis (Yang et al., 2015), and the collection of plants for this purpose may have had a negative impact on population sizes and genetic diversity, both now and in the past. Such effects have been reported in other medically important species in this region, such as *Rheum tanguticum* (Chen et al., 2009) and *Anisodus tanguticus* (Zheng et al., 2008).

### *Saxifraga banmaensis* should be treated as a synonym of *S. pasumensis*

Specimens of *S. banmaensis* were first collected by S.L. Chen in 2003 and later described as a new species, distinguished from *S. pasumensis* by the cartilaginous-mucronulate apex of the sepals and linear, clawless petals, compared to the acute sepals and pandurate to pandurate-oblong, clawed petals of *S. pasumensis* (Pan et al., 2006). However, our preliminary morphological study of type material, described in the Materials and Methods, cast doubt on whether these distinctions could hold up. Indeed, good evidence for a merger of the two taxa comes from our molecular phylogenetic analyses. For example, the cpDNA dataset indicates that the unique haplotype H1 found in *S. banmaensis* clusters with *S. pasumensis* haplotypes, with a high level of statistical support. Also, the *F*_*ST*_ values between *S. banmaensis* and the populations of *S. pasumensis* s.s. revealed a negative value for cpDNA data (−0.2919), indicative of an absence of differentiation. For the ITS dataset, one of the two genotypes of *S. banmaensis* (G1) clustered with those of *S. pasumensis*, but the other (G3) clustered with those of *S. umbellulata*, although support for this position was weak (Figure 2). The *F*_*ST*_ value (0.4525) between *S. banmaensis* and populations of *S. pasumensis* s.s. is comparatively small, suggesting only limited differentiation. Overall, it seems advisable to regard *S. banmaensis*, as a geographically marginal population synonymous with *S. pasumensis*.

### *Saxifraga pasumensis* should be treated as a separate species

Pan (1992) treated *S. pasumensis* as a variety of *S. umbellulata*, namely *S. umbellulata* var. *pectinata* (C. Marquand and Airy Shaw) J-T. Pan. This treatment was also adopted by Pan et al. (2001), but two co-authors, R.J. Gornall and H. Ohba, suggested that the variety would be better regarded as a separate species (Pan et al., 2001). There is no doubt that two taxa are involved. Firstly, the two are easily and consistently distinguishable by the margin of the basal leaves, which is cartilaginous setose-ciliate in *S. pasumensis* but glabrous/entire in *S. umbellulata*. In addition, phylogenetic reconstruction based on the cpDNA dataset shows two reciprocally monophyletic lineages, corresponding to the same two taxa, and showing a high degree of genetic differentiation. A very similar picture is shown by the ITS dataset, genotype G2 notwithstanding. Support for two clades is further emphasized by significantly high *F*_*ST*_ values (0.9393 for cpDNA; 0.7567 for ITS), suggesting considerable genetic divergence between the two taxa, consistent with strong reproductive isolation. The reproductive isolation is due at least in part to the largely allopatric distributions of the two taxa. Although some of the populations from the two species are geographically close, there is little evidence of hybridization between them. Finally, molecular clock analysis indicates that infraspecific divergence in *S. pasumensis* occurred much earlier than that in *S. umbellulata*, implying that the respective populations have had relatively long and independent evolutionary histories. Of course, assignment of rank is an arbitrary process but, in this case, the evidence is indicative of two well defined and distinct evolutionary lineages, with associated reproductive isolation, a pattern of variation consistent with the recognition of two species: *S. pasumensis* and *S. umbellulata*.

## Author contributions

QG conceived and designed the experiments, analyzed the data, wrote the paper, prepared figures and tables. YL, ZG, and LJ performed the experiments, analyzed the data. RG contributed distributional data and reviewed and wrote parts of the paper. JW and HL collected samples and analyzed the data. SC conceived and designed the experiments. QG and YL have contributed equally to this work.

### Conflict of interest statement

The authors declare that the research was conducted in the absence of any commercial or financial relationships that could be construed as a potential conflict of interest.
